# Biocompatibility and biodegradability of polyacrylate/ZnO nanocomposite during the activated sludge treatment process

**DOI:** 10.1371/journal.pone.0205990

**Published:** 2018-11-01

**Authors:** Chao Zhu, Huiqin Wang, Zarak Mahmood, Qing Wang, Hongrui Ma

**Affiliations:** Department of Environmental Engineering, College of Environmental Science and Engineering, Shaanxi University of Science and Technology, Xi'an, Shaanxi, China; Institute of Materials Science, GERMANY

## Abstract

Polymer nanocomposites have been widely used in the preparation of coating agents in the leather industry. Considering the complex bio-effect and ecological risk of nanomaterial, research on the biodegradability and biocompatibility of polyacrylate/metal nanocomposites in activated sludge system is of specific significance. We investigated the change of the bacterial community and diversity of activated sludge fed with polyacrylate/ZnO nanocomposites in both low (500 mg COD /L) and high (1000 mg COD/L) doses. COD removal, dehydrogenase activity, and total protein of activated sludge system were monitored in all treatments over 30 days. The results demonstrated that the exposure to both polyacrylate/ ZnO nanocomposite and polyacrylate decreased the dehydrogenase activity at early stage comparing to the control, but had no significant influence on COD removal. Overall, the bacterial communities in activated sludge were resistant to polyacrylate/ZnO nanocomposites, and had higher levels of metabolic activity, protein accumulation and bioavailability when exposed to these chemicals comparing to the control especially at later stages of incubation (15–30 days), which indicated that these polyacrylate/ZnO nanocomposites of certain amount were biocompatible to activated sludge system.

## Introduction

Nanoparticles have quantum and surface effect because of their small size, large specific surface area, and many unique physicochemical properties, making them a promising means of improving traditional materials and industries [1–3]. At the present, the development and application of artificial nanocomposites is one of the most attractive fields in research [4–7]. With the development of nanotechnology, from the laboratory to mass production, the environmental exposure and ecological risk is inevitable. The application of nanocomposites in the traditional tanning process is also being explored with success. Polymer nanocomposites use nanoparticles as a filler to improve traditional leather products resulting in greater strength, hardness, toughness, and anti-aging properties [8–10]. Various nanocomposite leather chemicals are prepared by initiating the production of free radicals on the surface of inorganic nanomaterials such as montmorillonite, SiO2, TiO2, and ZnO followed by polymerization of the vinyl or acrylic monomers in the presence of the free radicals [11–14]. It was reported that the addition of ZnO into the polymer matrix increases the thermal stability, dielectric properties, and wear resistance of the composites, making these nanocomposites potential applications in the fields of electronics, optoelectronics, energy storage and conversion, biomedicine, etc [15, 16].

In addition to their degradation and potential toxicity, polymer nanocomposites also have nanoscale effects and produce products that can interact with other pollutants, resulting in new pollution mechanisms, therefore it is important to clarify the ecological risks of polymer nanocomposites. Activated sludge system is the principle carrier for any new chemical before its release to the environment. A proper assessment of its ecological risk primarily requires an understanding of the physical and chemical changes that polymer nanocomposites can cause in wastewater treatment. In addition, the migration, physical, and chemical behavior of nanocomposites may have a significant impact on microbial communities and function. Up to the present, studies on the environmental risks of nanocomposites have focused mainly on their impact on terrestrial and aquatic ecosystems, and relatively few systematic studies have been conducted in sewage systems [17–19].

The mechanisms of nanomaterial toxicity can include the adsorption of the nanomaterial to the surface of microbial cells induced by reactive oxygen species, lipid peroxidation, protein denaturation, and DNA damage, as well as the toxic effects of nanometal oxide oxidation [20, 21]. Polyacrylate tanning agents are widely used in the leather industry and differ widely in their degradability because of the fact that different monomers such as acrylic acid, methacrylic acid, acrylonitrile, acrylamide, styrene and maleic anhydride are used for polymerization [22, 23]. The toxicity of degradation intermediates of methacrylic polymers depends on their method of synthesis, such as whether metal oxide nanoparticles were introduced into the methacrylic polymers or not; the question whether this increases the toxicity of methacrylic polymers requires further study [24]. The destruction of microbial communities and their enzymatic activities in activated sludge could potentially endanger the ecological functions of microorganisms and adversely affect the related wastewater treatment and ecosystem health [25]. Therefore, a study to investigate the biocompatibility and biodegradability of these chemicals in activated sludge is necessary to evaluate bio-safety, especially considering that some of these chemicals are referred to as being environmentally friendly products but have not been properly evaluated. The lack of relevant published studies in this area makes our work imperative.

In the present study, we aimed to determine if polyacrylate latex, or its ZnO nanoparticle composite, act to affect the bacterial community in activated sludge rather than just its ecological function characterized by dehydrogenase activity, total protein content and COD (chemical oxygen demand) removal performance. The novelty of this study is to take the activated sludge from MWTP as an ecological suffering system to evaluate the biocompatibility and biodegradability of advanced polymer/metal nanocomposites and to elucidate whether bacterial community success will cause concomitant change on metabolic activity under such like pressure.

## Materials and methods

The activated sludge sample was collected from the aerobic tanks of a cyclic activated sludge system (CASS) process in a full scale municipal wastewater treatment plant (MWTP) in Xi’an (N 34°38´20.89″ E 109°01´14.47″), China during October 2010. These samples were authorized by Xi'an Eureka Environmental Technology Co., Ltd for the approval of this study. The main characteristics of the original sludge are TSS (total suspended solids) 3.5 ± 0.27 g/L, VSS (volatile suspended solids) 3.03 ± 0.21g/L, total protein 3.65 ± 0.16 mg COD/L, total carbohydrate 2.32 ± 0.15 mg COD/L, and pH 7.00 ± 0.05. The polyacrylate/ZnO nanocomposite [26] tested here were acquired from the factory and directly fed to the activated sludge without any pre-treatment.

**Fig 1:**
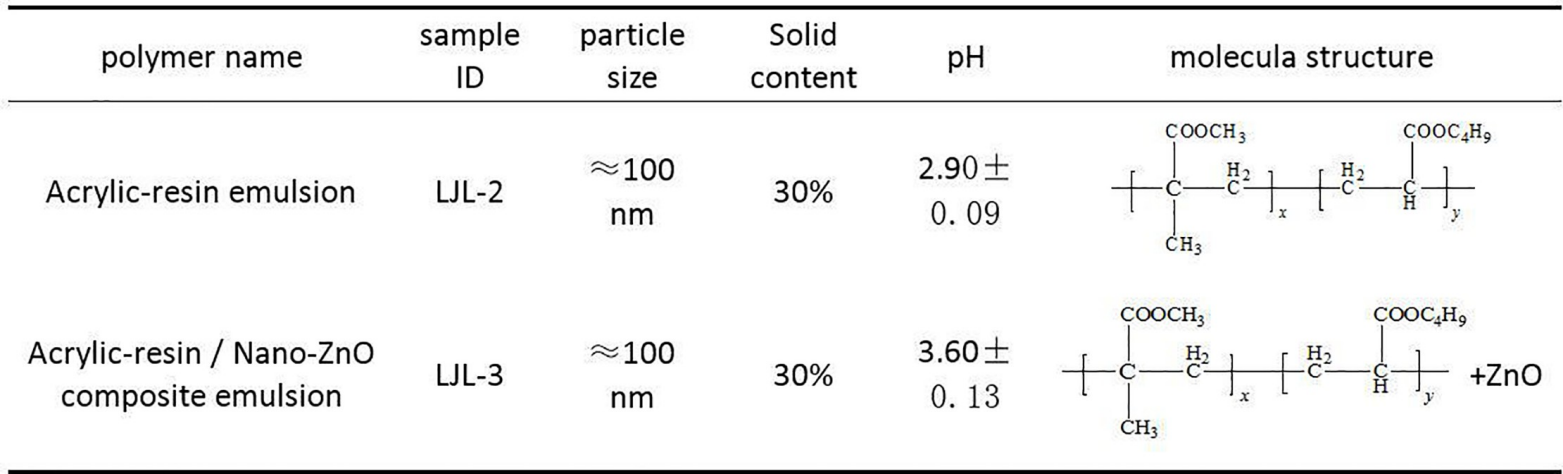
Descriptions of the testing polyacrylate-polymer prepared coating agents.

A cylindrical polypropylene container set in a water bath of constant temperature constituted the lab-scale treatment system without sludge return. An air pump was used in conjunction with a gas-flow meter to introduce air into the diffusers at the bottom of the aeration column to provide the designated DO (dissolved oxygen) level of 2~3 mg/L during incubation. Sterilized and deionized water was supplemented as needed to counteract any evacuation losses. To conduct the experiment, six treatments were prepared containing the inoculated sludge (at a TSS value of 1.67 g/L) obtained from a parent sequential batch reactor (SBR), which had been operated for over a week and had achieved a stable removal of biological nutrients (approximately 80% and 99% of nitrogen and phosphorus removal). Afterward, four of the treatments were fed with LJL-2 and LJL-3 at two different doses to achieve two COD levels of 500 and 1000 mg/L for each addition (see Fig 1). Treatment with the addition of glucose (1000 mg COD/L) was used as the reference treatment, and no glucose addition was used as the control treatment. Finally, deionized water was added to produce a working volume of 5 L. Each treatment was performed three times, cultured under normal lighting conditions (about 12 hours of light and 12 hours of darkness), maintained at 30 ± 1°C, and aerated for 1.5 hours three times a day. The operating time was recorded when the test coating agent or the glucose was added. The sludge was collected from the culture at 0 and 12 hours and at days 1, 2, 5, 10, 15, 20, and 30 for the measurement of COD, dehydrogenase activity, and protein concentration. The initial and final sludge samples were collected, morphologically characterized, and assessed for interference using a light microscope.

### DNA extraction

Genomic DNA was extracted from triplicate samples fed with LJL-2 and LJL-3 at high doses on days 5 and 20 using a Fast DNA spin kit for soil (Q-Biogene, Solon-Ohio, Burlingame-California, USA), according to the manufacturer’s instructions. DNA integrity was assessed by 0.8% agarose gel electrophoresis. The purity and the quantity of the extracted DNA was determined by UV spectrophotometry at 260 and 280 nm. DNA extracts were stored at -20°C.

### DGGE analysis

The partial V3 regions of bacterial 16S rRNA genes were PCR-amplified from the genomic DNA using the primers 338f-GC (5'- CCT ACG GGA GGC AGC AG -3' with a GC clamp attached to the 5' ends of the primer) and 518r (5'- ATT ACC GCG GCT GCT GG -3') [27]. PCR amplification was carried out in a total volume of 25 μL containing 10 ng of template DNA, 1 × Ex Taq reaction buffer, 1 U Ex Taq polymerase, 1.5 mm MgCl2, 0.2 mm dNTPs, and 0.5 μM primers using a T-gradient thermocycler (Biometra, Göttingen, Germany). The amplification profile consisted of an initial denaturation step at 94°C for 5 min, 30 cycles of denaturation at 94°C for 1 min, annealing at 55°C for 45 s, and extension at 72°C for 1 min, followed by a final extension at 72°C for 10 min. PCR products were electrophoresed on an 8% polyacrylamide gel in 1×TAE buffer with a gradient ranging from 35% to 55% denaturant (100% denaturant: 7 M urea and 40% (v/v) deionized formamide) at a constant voltage of 160 V for 5 h at 60°C using a DCode Universal Mutation Detection System (Bio-Rad, U.S.A). The gel was silver-stained for 15 min and visualized with a Gel-Doc2000 system (Bio-Rad, U.S.A). All detectable bands (labelled 1–41) were then excised from the gel, and after clean-up treatment using Poly-Gel DNA Extraction Kit (OMEGA, U.S.A) the recovered DNA was amplified (initial denaturation at 94°C for 4 min, 30 cycles of denaturation at 94°C for 30 s, annealing at 55°C for 30 s, and extension at 72°C for 30 s, followed by a final extension at 72°C for 10 min), purified, cloned into pMD18-T vector (TaKaRa, Kusatsu, Shiga 525–0058, Japan) and sequenced using an ABI PRISM 3730 automated DNA sequencer (Applied Biosystems Inc, 850 Lincoln Centre Drive Foster City, USA). Chimeric sequences were identified using the CHIMERA CHECK program in RDP (Ribosomal Database Project) and excluded from subsequent analysis [28]. All sequences obtained were aligned with those of the 16S rRNA genes deposited in the GenBank database using the Blast search program of NCBI to find the closest related species. All sequences were assigned to the genus level grouping with 90% confidence by the “Classifier” program in RDP [28]. The 16S rRNA gene sequences obtained in this study have been submitted to the GenBank database under the accession numbers KC812054 to KC812061, KC812063 to KC812070, KC812072 to KC812080, KC812083 to KC812085, and KC812087 to KC812099.

### Analytical methods

DO and pH measurements were conducted using a Professional Plus DO-meter (Yellow Springs, Ohio 45387–1107) and a pH-meter (Jingke Shanghai, China), respectively. The determinations of COD, TSS, and VSS were conducted in accordance with the standard methods [29]. Total protein and dehydrogenase activity were measured using the Coomassie Brilliant Blue G250, and triphenyltetrazolium chloride (TTC) methods, respectively [30, 31]. Infrared spectroscopy of the sludge before and after incubation was obtained using a Bruker Vector 22 FTIR (Bruker, Berlin, Germany) to observe changes in functional groups to assess the biodegradation of the polyacrylate/ZnO nanocomposite and the possible mechanism.

### Data analysis

An assessment of the similarity of DGGE bands in the 16S rRNA gene profiles was performed using both Jaccard’s coefficient and the comparison tools in Quantity One Gel software. To compare the bacterial diversity for each treatment, we analyzed the optical densities of each detectable band in the DGGE gel using Quantity One software (Bio-Rad, Hercules, CA, USA), combined the phylogenetic analysis, and then calculated the Shannon-Wiener index and the Jaccard similarity matrix [32, 33]. Pianka and Levins indexes were also calculated for niche overlap and niche width evaluation to show how intense the competition was among different bacterial communities in the activated sludge during the treatment process [34]. In addition, a canonical correspondence analysis (CCA) of the bacterial community composition based on the DGGE bands for different treatments was used to compare the influence of exposure dose and time on bacterial community structure [35]. CCA was performed using Conoco for Windows 4.5. (Canoco: software for visualization and multivariate analysis, Wageningen Plant Research, PO Box 16, 6700AA WAGENINGEN).

## Results

### Performance of treatment with the polyacrylate coating agent on a lab scale

**Fig 2:**
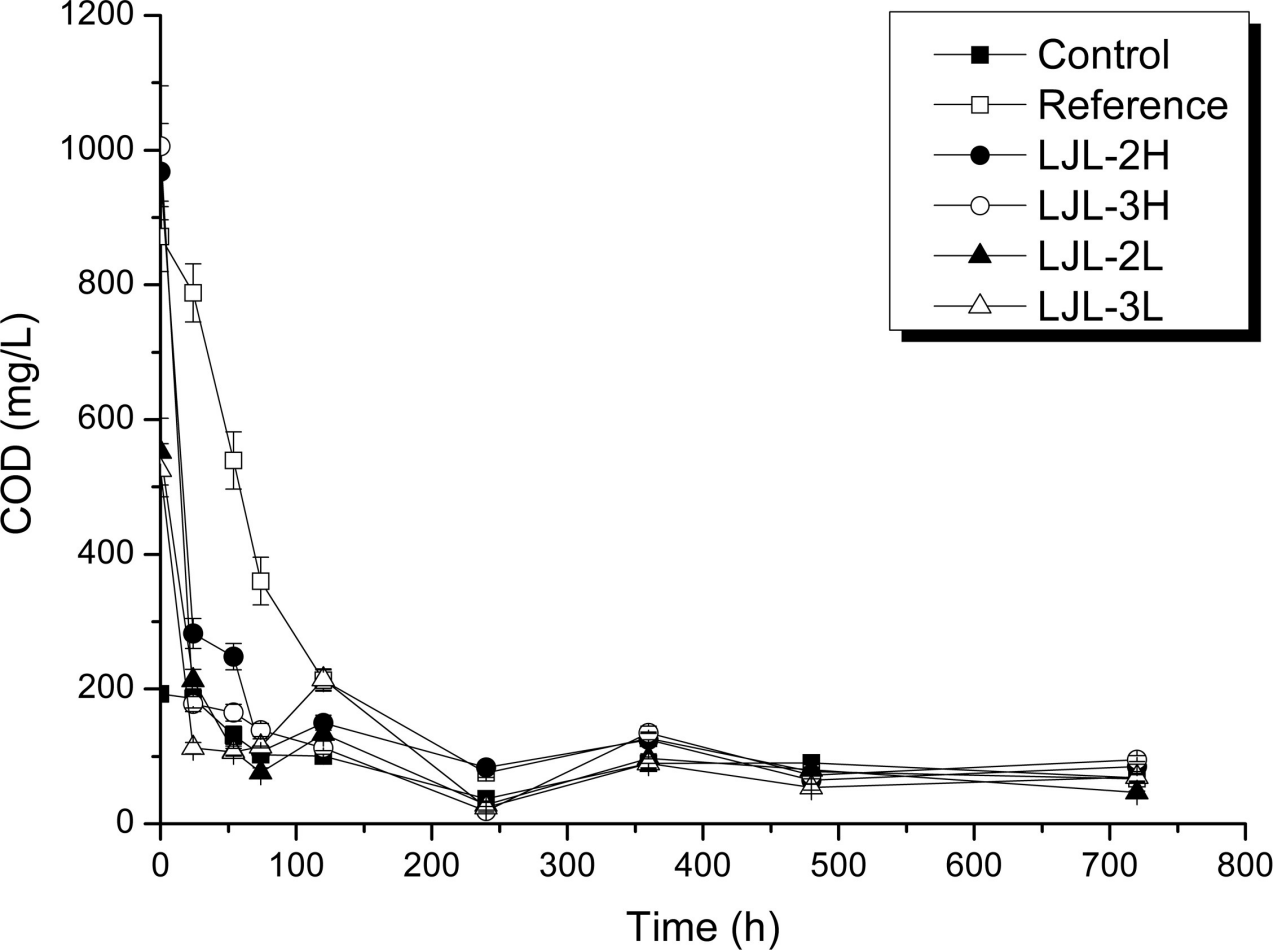
Changes in the COD Concentrations in the activated sludge incubations fed with different coating agents at different doses. The error bars (Standard deviation) are derived from three replicates. The two acrylic polymer coating agents were LJL-2 and LJL-3, with ‘H’ referring to a high dose of 1000 mg/L and ‘L to a low dose of 500 mg/L. The incubation with only the original activated sludge was set as the control while the incubation with the addition of glucose was reference.

Fig 2 shows the changes in COD concentration during 30 days of continuous operation of the lab-scale system. The DO and pH values basically remained stable, ranging from 2.01~2.25 mg/L and 6.87~7.01 mg/L respectively. Under these conditions, COD concentration dropped to a steady, low level (< 200 mg/L) after 10 days of incubation regardless of treatment. Except for the reference incubation, the highest COD removal rates for all treatments were observed after 24 h and then remained constant at a low level in the rest days. Interestingly, no significant differences in COD removal were detected in treatments fed with the polyacrylate latex, or its ZnO composite, indicating that absorption, at least at the beginning, is the possible mechanism for their COD removal. In the first five days, the COD of the reference had the expected higher COD level compared to the other treatments, then COD concentration in all the samples were stable at a similar level, indicating no adverse effect was caused by the exposure of polyacrylate/ZnO nanocomposite to activated sludge system.

**Fig 3:**
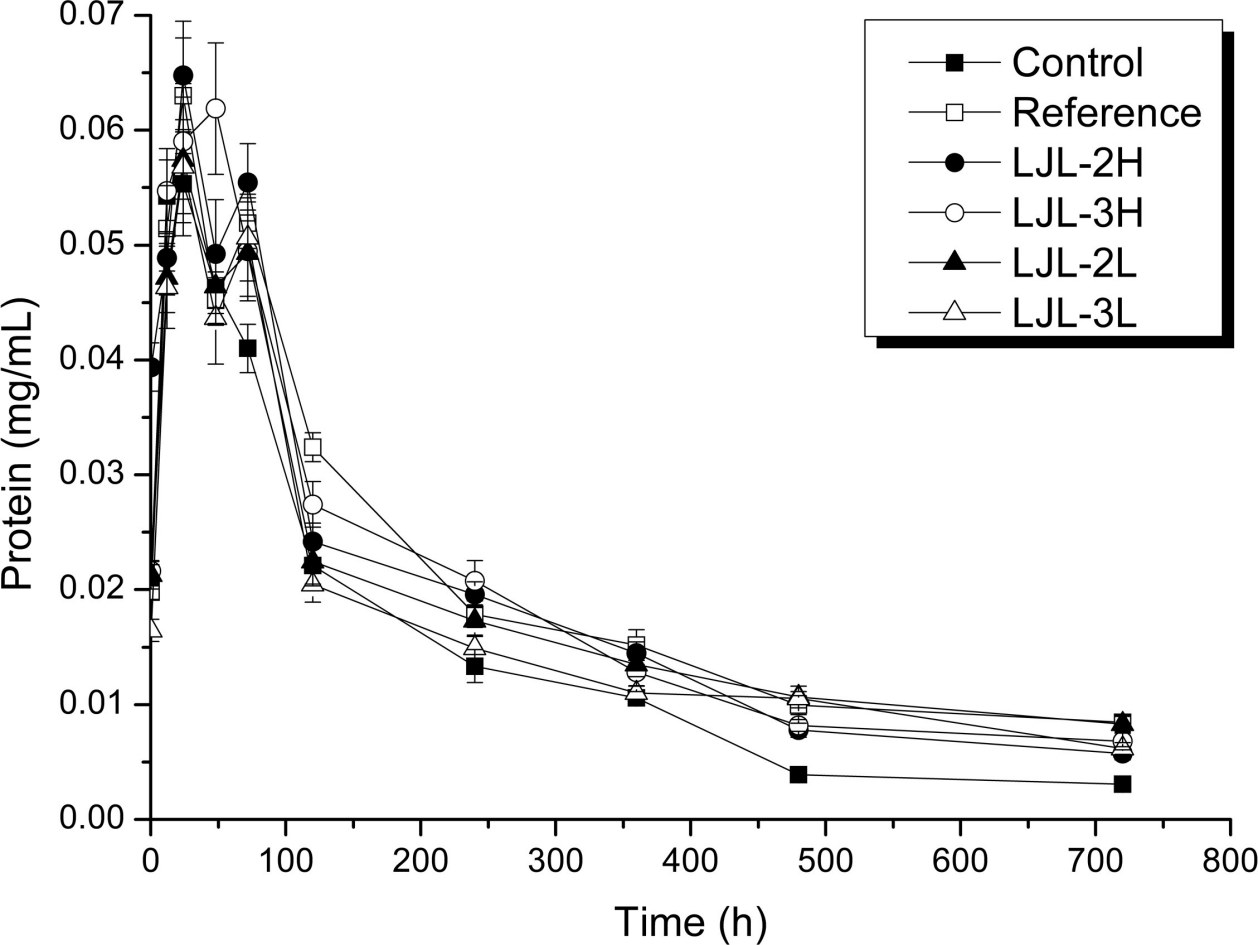
Changes in the protein content in the different activated sludge incubations. The error bars represent the (standard deviation) of the mean of three replicates.

Protein levels and dehydrogenase activity were measured beginning 0.5 h after incubation. As shown in Fig 3, the protein concentration in all treatments generally decreased during incubation, with some fluctuations observed over the first 3 days. Except for the control, the protein content in all the treatments increased within 24 h, LJL-2H, increased by 60%, being the lowest, and LJL-3L, which continued to grow until day 2, having the greatest increase. The protein content in the reference, LJL-2H, LJL-2L, and LJL-3L treatments showed a "V" pattern from day 1 to day 3. Clearly, the final protein levels in the polyacrylate latex treatment, or its ZnO composite, as well as the reference, were higher than that of the control, indicating the bioavailability of polyacrylate/ZnO nanocomposite to activated sludge system is similar to glucose at least in protein growth.

**Fig 4:**
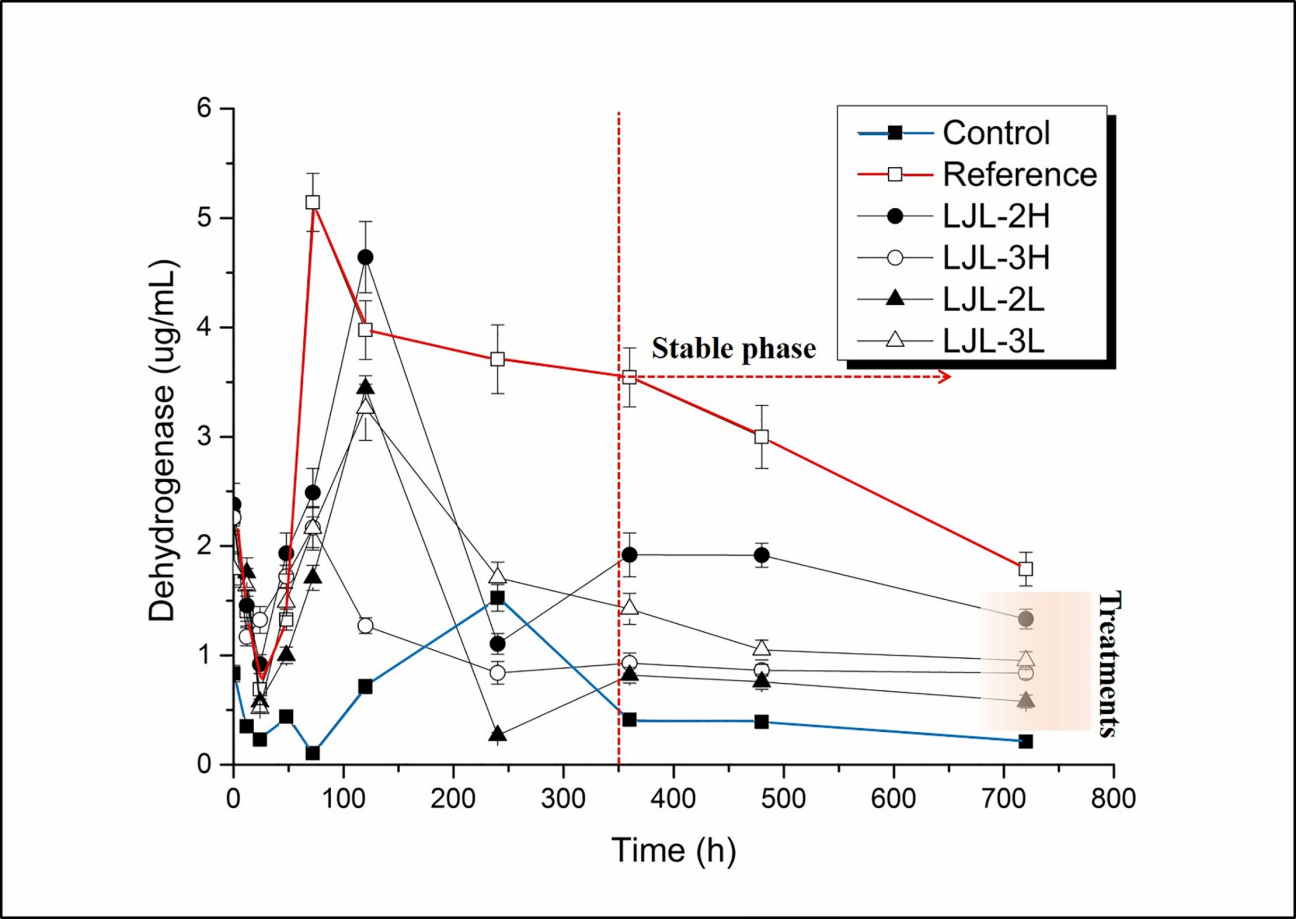
Changes in the dehydrogenase activity levels in the different activated sludge incubations. The error bars represent the (standard deviation) of the mean of three replicates.

Dehydrogenase activity change reflects the effect of the polyacrylate nanocomposite on the microbial metabolism of organic matter in the activated sludge. As shown in Fig 4, the treatments fed with the polyacrylate latex, or the polyacrylate/ZnO nanocomposite, generally had higher dehydrogenase activity compared to the control, but both had lower activities compared to the reference. The changes of overall dehydrogenase activity levels were similar between LJL-3L and LJL-3H, whereas LJL-2L and LJL-2H had similar changing trend but the absolute value were significantly different from each other, which indicates that exposure does contributes more to the bio effect of polyacrylate nanocomposite on activated sludge. This can be explained as the stressing effect caused by increased ZnO concentration. The dehydrogenase activities showed decreases to different degrees (150%~679%) in the first 24 h, except for LJL-3H where the dehydrogenase activity started to increase from 12 h after a prior decrease of 93%. After 24 h, the dehydrogenase activity levels in all treatments increased, gradually or sharply, from 3 days for the reference, LJL-3H, and LJL-2L to 5 days for LJL-3L and LJL-2H and finally 10 days for the control. Then the dehydrogenase activity levels in all treatments started to decrease till the end of the incubation, the dehydrogenase activity levels in treatments were right between that of the control and reference. It was obvious that polyacrylate/ZnO nanocomposites caused no inhibition to microbial metabolism comparing to starving.

Differences in the microbial metabolic activity, which represents the ratio of dehydrogenase activity to the total protein concentration in each treatment, could be clearly observed with the different treatments (see S2 Fig). The activities at the end of the incubations were higher than at the beginning. The greatest increase was observed for the control (959%), followed by LJL-2H (777%), and the reference (776%).

**Fig 5:**
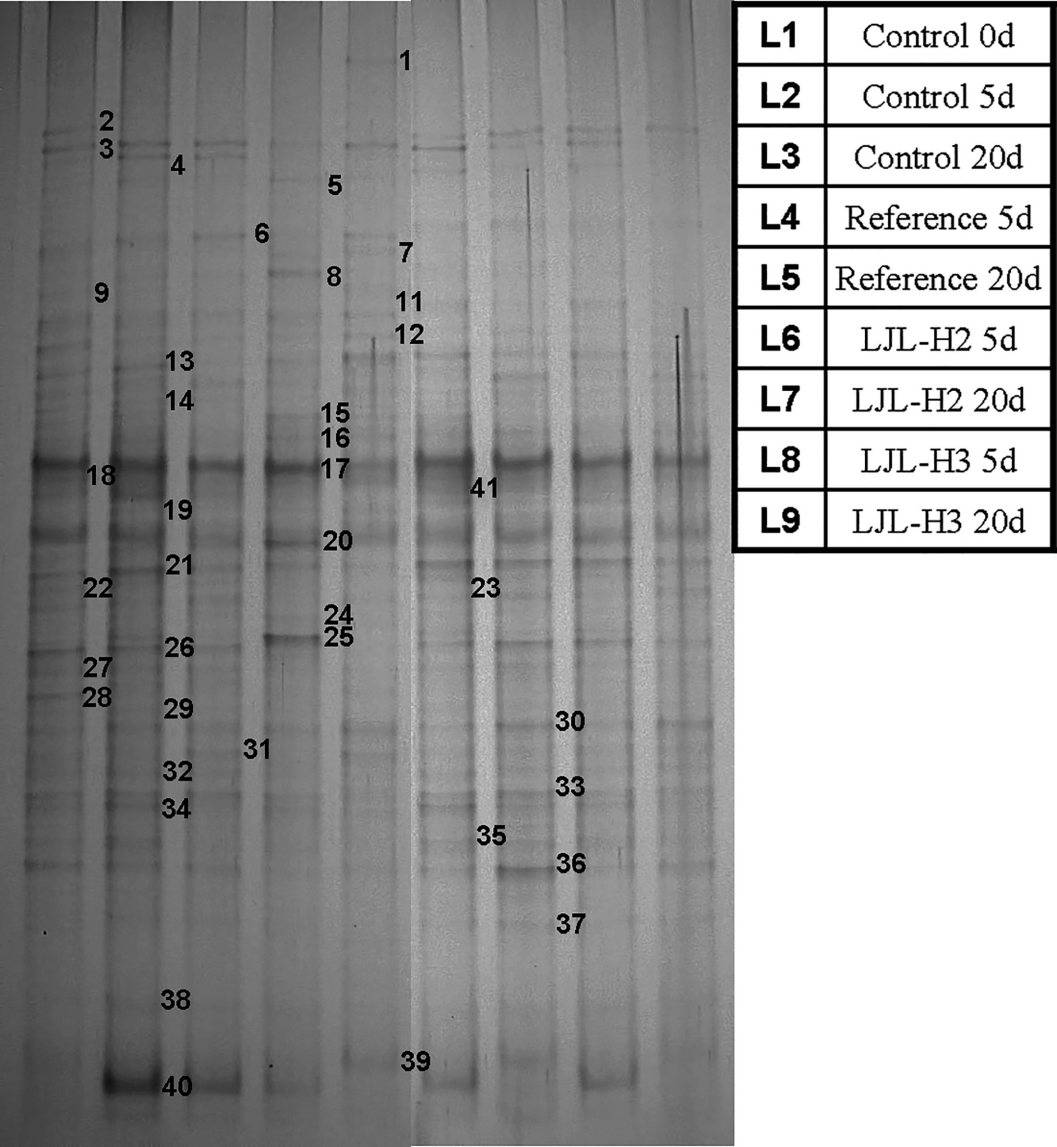
DGGE analysis of the partial V3 variable region of bacterial 16S rRNA genes amplified from DNA isolated from the activated sludge community. The band numbers correspond to the sequences shown in the table affiliated to Fig 4. Lanes (from left to right) are numbered L1 to L9, and their referencing samples are displayed in the side content table (0d, 5d, 20d represent different sample times where ‘d’ means day).

**Fig 6:**
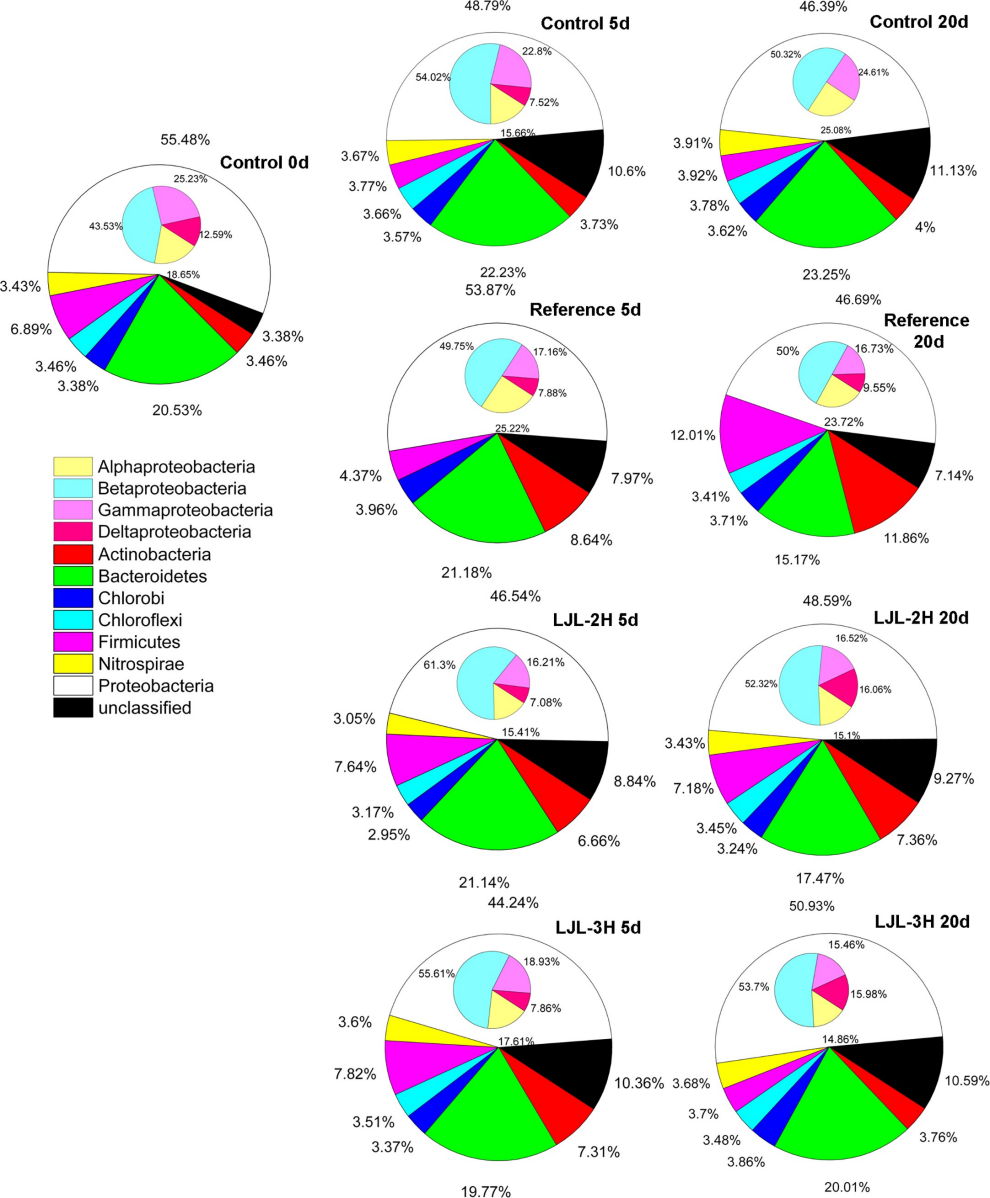
Composition of the bacterial community and succession based on the principle phyla present in each activated sludge sample based on a DGGE analysis and sequencing. Details of the grouping used for the different ribo-types in the general and class levels are shown in S1 Table. The abundance of a given species is shown as the percentage of its optical density in DGGE gel.

**Fig 7:**
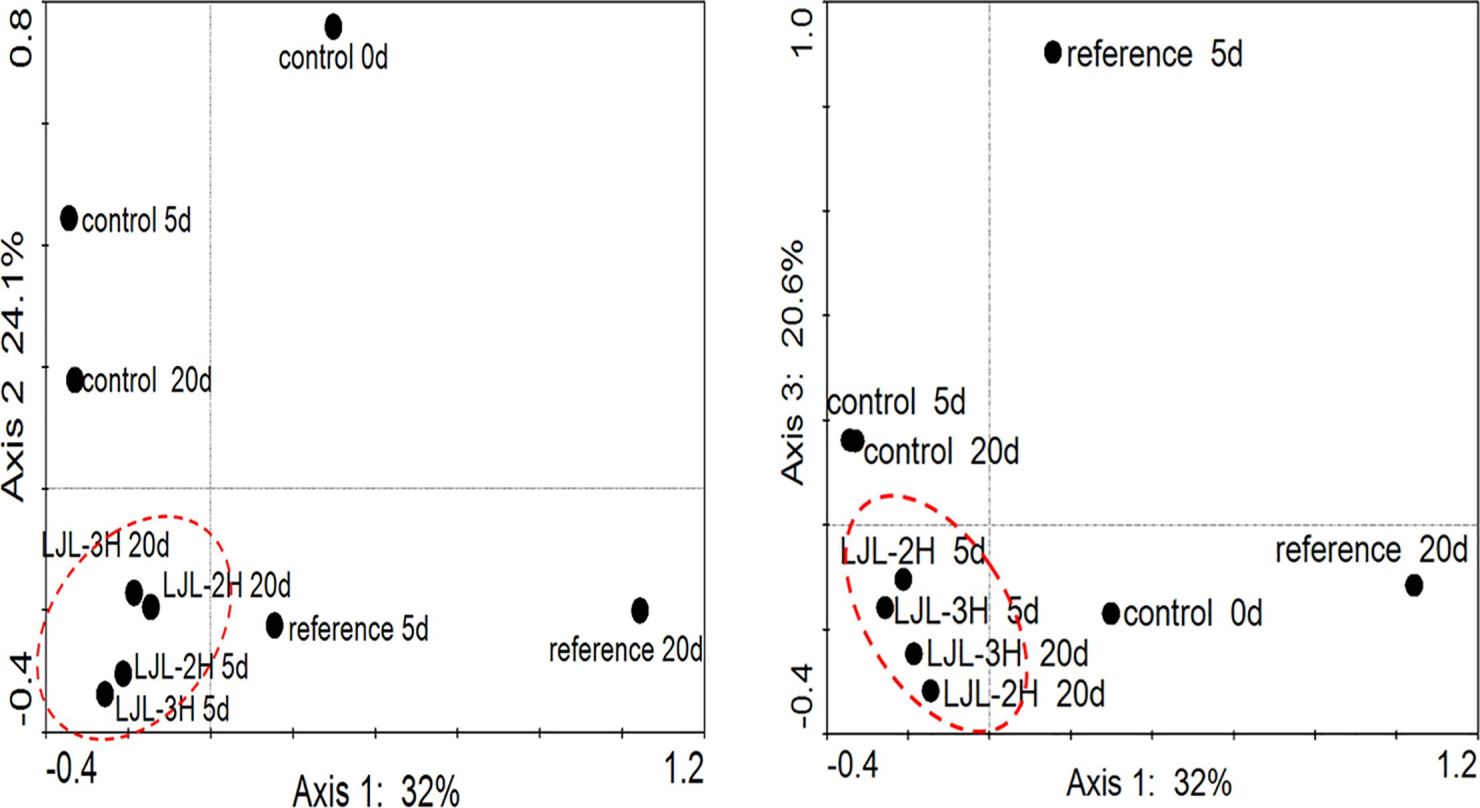
Canonical Correspondence Analysis (CCA) of the composition of the bacterial community composition in the different treatments based on the DGGE bands.

Sequencing of different 16S rRNA genes allows for an investigation of the spatial and temporal changes in the population in any environment, and also allows for the identification of the predominant species in a community [32]. In our study, to examine how the polyacrylate/ZnO nanocomposite affects the bacterial community and which dominant bacterial species are found in the activated sludge samples, the V3 variable regions of the bacterial 16S rRNA genes were amplified by PCR from the community DNA and subsequently analyzed by DGGE. The bands labeled 1–41 on the DGGE gel shown in Fig 5 were sequenced. The closest matches for the resulting sequences were then determined using a Blast search of GenBank (see S1 Table). *Dechloromonas aromatica*, *Clostridiales* sp., and *Acinetobacter oleivorans* were the most stable and dominant populations based on the densities and distribution of their bands. Some treatment-specific or treatment-adaptive species were also observed including *Exiguobacterium antarctica* in the initial sludge, *Frateuria* sp. and *Bdellovibrio bacteriovorus* in the reference treatment at 5 d, *Bifidobacterium adolescentis* in the reference treatment at 20 d, and *Sorangium cellulosum* in the initial sludge at 5 d. Statistically, most of the sequences could be assigned to five types of bacteria: *Betaproteobacteria*, *Gammaproteobacteria*, *Alphaproteobacteria*, the *Bacteroidetes*, and unclassified bacteria [36] which are ubiquitous in wastewater treatment systems (see Fig 6). Some potential dominant genera, such as *Firmicutes*, *Deltaproteobacteria*, *Actinobacteria*, *Chlorobi*, and *Nitrospirae*, were found in some or all of the samples. Based on the DGGE profile, we noted that incubation time caused a small divergence in the sludge bacterial community from the initial one (see Fig 7). LJL-2 or LJL-3 radically increased this degree of dissimilarity (see S2 Fig). However, the similarity of the structure of bacterial community between samples indicated that there was no specific relationship between the different additives or incubation time and their effect on bacterial communities, so we could conclude that introducing the ZnO nanoparticles to the polyacrylate latex has no unique influence on the bacterial community.

### Bacterial community diversity and succession

The original sludge had the highest bacterial biodiversity (see S3 Table), which then decreased over time. The addition of a high concentration of LJL-2 did not decrease the sludge bacterial diversity, which remained close to the original sludge even after 5 d of incubation. However, in the case of LJL-3, its addition at a high concentration for 5 d decreased the diversity in the sludge bacteria diversity to a level close to the original sludge after 20 days of incubation. In contrast, compared to the control and LJL-2, the bacterial diversity in the LJL-3 treated sludge tended to increase, although only modestly.

Fig 6 shows the succession of the major populations in the different treatments during the 20-day incubation. With the largest niche width (see S4 Table), *Proteobacteria* was the absolutely dominant population in all treatments, ranging from 44.24% in LJL-3H at 5 d to 55.48% in the initial sludge, while the size of its population decreased to different extents during the incubation. The size of the population of unclassified species, compared to the initial sludge (3.38%), showed a large increase in all the treatments once the incubation began, ranging from 7.14% to 11%. Other obvious changes could be detected in the other populations: proliferation of *Actinobacteria* in the reference, LJL-2H, and LJL-3H treatments; the expansion of *Bacteroidetes* in the control treatment; the complete replacement of *Nitrospirae* with other populations was only observed in the reference treatment.

### Infrared spectroscopy analysis

**Fig 8:**
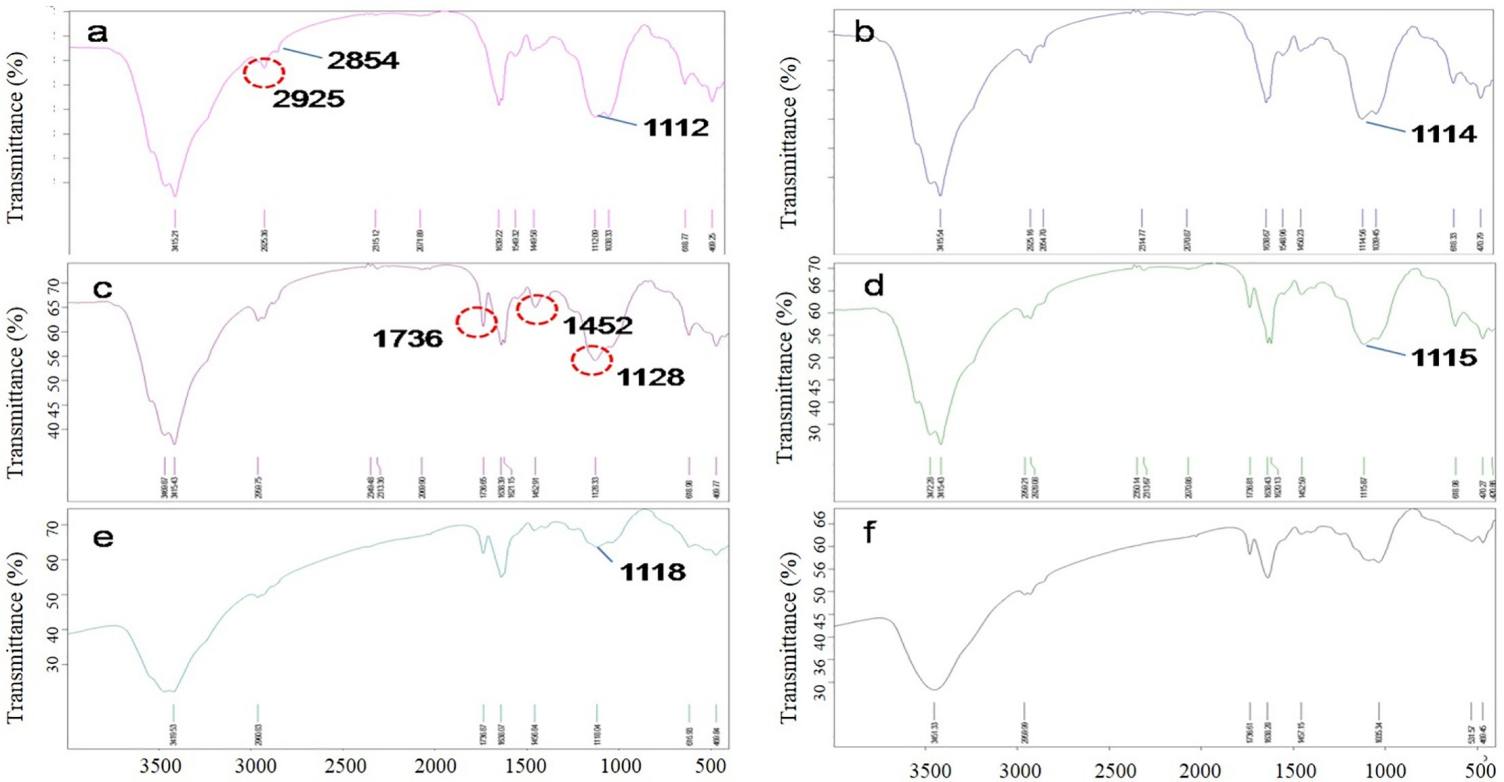
Infrared spectroscopy of the different sludge treatments after 30 days of incubation. (a) initial sludge; (b) sludge fed with glucose (1000 mg COD/L); (c) sludge fed with LJL-2 (1000 mg COD/L); (d) sludge fed with LJL-3 (1000 mg COD/L); (e) sludge fed with LJL-2 (500 mg COD/L); (f) sludge fed with LJL-3 (500 mg COD/L).

Fig 8(A)–8(E) shows the changes in the infrared spectra of the activated sludges from the different treatments. The assignments of the various bands and peaks made in this study are in reasonable agreement with those reported in the literature for similar functional groups [37–39]. The initial sludge and the sludge fed with glucose had the same infrared spectroscopy profile; the bands at 2925 and 2854 cm−1 are, respectively, due to the C-H stretching absorbance of a methyl group and of a methylene group. The strong band at 1736 cm-1 indicates carbonyl stretching, a vibration that was only observed in the treatments with the polyacrylate latex and its ZnO nanocomposite and was weaker at lower doses, indicating degradation had occurred. The band at 1453 cm-1 corresponds to the -COO stretching vibration. The band at 1128 cm-1 in Fig 7(C) can be attributed to C-O stretching, which shifted from 1110 to 1122 cm-1 in the other treatments. As shown in Fig 7C–7F, the introduction of ZnO to the polyacrylate latex decreased the stretching vibration of C = O as was clearly shown by the 1736 cm-1 band, which is more obvious in higher concentration, suggesting that the ZnO nanoparticle inhibits the degradation of the polyacrylate/ZnO nanocomposite. Interestingly, there was no characteristic Zn-O band at 483 cm−1, or its red shift, as previously described [40], in the treatments with the polyacrylate/ZnO nanocomposite, which suggests a possible transformation of the ZnO.

## Discussion

Currently, activated sludge is an essential, key factor, during most of the wastewater treatment processes in a WTP. Sludge is defined as being a very heterogeneous group of microorganisms, dominated by a series of bacterial taxa [41]. Therefore, any chemical or physical disturbance of the bacterial community will influence the entire wastewater treatment system evaluated by its COD elimination efficiency, which consists of nitrification, denitrification, and the removal of phosphorus [42–44]. It is especially important to investigate how changes in the structure of the bacterial community occur in response to chemicals released in the wastewater treatment system over a period of 5 days, since the residence time of wastewater in a MWTP is not usually more than 5 days. Like BOD5 (Biochemical Oxygen Demand after 5 days), a 5-day bacterial community and metabolic activity can also be considered a measure to assess biodegradability and possible toxicity during the handling and processing of industrial chemicals.

After 5 days of exposure to a single addition of a latex polyacrylate, or its ZnO nanocomposite, at both high and low COD levels (500 and 1000 mg COD/L), the protein levels were not found to be altered significantly (range of 0.0204 to 0.0274 mg/mL) compared to the initial sludge (0.0213 mg/mL), which reflects the stability of the sludge over this period. In addition, after 30 days of exposure, in general, there was greater accumulation of protein than the control but at very low levels (0.0058 to 0.0083 mg/mL) compared to the initial sludge. However, there was no specific effect on the protein content because of the introduction of ZnO nanoparticles to the polyacrylate. The metabolic activity, assessed by dehydrogenase activity in this study, was clearly higher on the fifth day of exposure (1.2712 to 3.9760 mg/ml) compared to the control (0.7154 μg/ mL). Due to the observation that the ZnO-filled polyacrylate had a lower activity than the ZnO-free polyacrylate, particularly at a high concentration, this suggests some negative influence on the sludge bacteria because of the presence of the ZnO nanoparticle. At the end of the incubation, the absence of the Zn-O band in the infrared spectroscopy analysis and the low final dehydrogenase activity seen at the high dose of ZnO-filled polyacrylate suggests that the entire ZnO nanoparticle could have been released as Zn2+, which could suppress bacterial growth to practically zero according to a previous observation in soil [45]. This hypothesis is consistent with a report that there is rapid dissolution/transformation of ZnO to an adsorbed phase of Zn (suspension of kaolin pH 7) within 24 h [46], and therefore, the dissolution of ZnO nanoparticles in the activated sludge over a period of 30 days is not unexpected.

We did not determine whether Zn2+ was incorporated into bacterial cells, or adsorbed onto the surface of cells, or perhaps bound to small abiotic particles. Here, the possible toxic mechanism may be due to the interactions of the metal nanoparticles with reactive oxygen species or a direct production of hydrogen peroxide radicals [47, 48], indicating that oxygen stress may have played a role in bacterial ecotoxicity. The ionic strength, the pH, the redox state, the DOM (Dissolved organic material) characteristics, and light are all environmental factors that influence the aquatic levels of reactive oxygen species, which in turn influences the reactivity and toxicity of the nanoparticles.

The DGGE analysis showed that treatment with the polyacrylate latex, or its ZnO nanocomposite (1000 mg COD/L), at a lower taxonomic level like genus, diverged from the initial sludge community by up to 78.3%. After five days, compared with the control and the ZnO-free polyacrylate, the polyacrylate filled with ZnO nanoparticles did not cause an obvious change in the structure of the bacterial community at the highest exposure level (1000 mg COD/L). With respect to incubation time, the perturbed communities did not return to their original structures even after 20 days of observation. Four bacterial responses to exposure to polyacrylate latex, or its ZnO nanocomposite, were observed: (1) the populations decreased in scale (intolerant), (2) the populations were initially reduced in scale and then over time increased again (recovering or tolerant), (3) the populations increased in scale (potential degrader of the polyacrylate/ ZnO nanocomposite), and (4) populations that were rare in the negative control then increased with exposure (bioavailability). These results demonstrate for the first time the bioavailability of polyacrylate latex, and its ZnO nanocomposite, in activated sludge through an examination of bacterial communities, which is also verified by the higher final protein accumulation compared with the control. However, the COD determination indicates that absorbance, not biodegradation, is the main mechanism for their removal, at least during the first 3 days. The recovery of the COD values from the third day to the fifth day, and its consequent decrease in the treatments with polyacrylate latex, or its ZnO nanocomposite, suggest the possible release and the concomitant degradation of these chemicals, which was also observed over day 10 to day 20.

The addition of ZnO nanoparticles to the polyacrylate caused an increase in alpha, beta, and gamma Proteobacteria, as well as in unclassified populations, which may be caused by the weakening of other populations, confirming that Proteobacteria are population of great adaptability or high resistance to the nanocomposite in our study. The proliferation of the unclassified population likely reflects the emergence of a new species tolerant to the ZnO nanoparticle or to Zn2+ itself. Moreover, extracellular polymeric substances present in the activated sludge might have stabilized the ZnO nanoparticle, such that it remained associated with bacterial particles at high concentrations without a concurrent increase in toxicity. The healthy and similar sludge morphology (see S2 Fig), obtained by light microscope, in sludge fed with ZnO-free or ZnO-filled polyacrylate at 100, 500, and 1000 mg COD/L respectively, may help to explain this. Polyacrylates are generally resistant to degradation [49], whereas in our study the addition of polyacrylate latex caused a bacterial community succession more similar to the reference treatment than to the control (if the disappearance of *Nitrospirae* is ignored), indicating that the polyacrylate is bioavailable. The possibility that polyacrylate might directly influence bacterial communities because of its toxicity can be ruled out because of the ubiquity of short-chain carboxylic acids in natural aquatic systems. Consequently, the interpretation of the composition shift in the bacterial community in response to polyacrylate treatment is likely due to altered carbon availability in the environment [50]. For reference, this also explains the observation of convergent development (with lower biodiversity) in a bacterial community in the presence of single carbon resource (see Fig 6). Tolerant bacteria such as gamma-*Proteobacteria*, *Nitrospirae*, *Chlorobi*, *Cloroflexi*, the *Bacteroidetes*, and *Firmicutes* were not able to exploit the increases when exposed to ZnO-free or ZnO-filled polyacrylate and generally scaled down comparing to control (see Fig 7). The appearance and stimulation of unclassified species following exposure to a high concentration of ZnO-filled polyacrylate suggests that these bacteria can cope with the adverse conditions induced by the presence of the ZnO nanoparticle. There are a couple of possible reasons for this: first, the bacteria that benefited from the addition of the ZnO nanoparticle might contain resistance genes and second, some bacteria phylotypes were inactive, resting, or existed as spores at the time of exposure and were less affected [51].

## Conclusion

The present study demonstrated for the first time that there are observable changes in the bacterial community structures resulting from exposure to a coating agent made of polyacrylate/ZnO nanocomposite in MWTP activated sludge. The long-term convergent development in the bacterial community, the increase in dehydrogenase activity, and the final accumulation of protein suggest the possible biodegradation of the polyacrylate latex and its nanocomposite ZnO. However, from the incubation data take over the first 5 days, especially considering the normal treatment time (<5 d) in the typical MWTP, it could also be concluded that the addition of polyacrylate latex, or its ZnO nanocomposite, up to a high 1000 mg of COD/L did not influence the removal of COD nor radically change the bacterial community in the MWTP activated sludge, which is evidence for its adequate biocompatibility.

## Supporting information

S1 Table Sequence similarities of excised DGGE bands shown in Fig 5. (PDF)

S2 Table Jaccard similarity matrix of bacterial communities in all samples. (PDF)

S3 Table Bacterial community diversity of different sludge treatments. (PDF)

S4 Table Niche width of the main populations observed in incubations. (PDF)

S5 Table Niche overlap of the bacterial populations observed in activated sludge incubations. (PDF)

S1 Fig Comparisons of proportions of dehydrogenase to protein in activated sludge incubations on both 12th and 720th hour. The proportion was calculated by the averages of dehydrogenase and corresponding protein concentration.
(PDF)

S2 Fig DGGE fingerprint sketch to order the samples according to their similarities comparing with L1 which represents the bacterial community of original activated sludge. (PDF)

S3 Fig Microscope images of the sludge treatments after 30-day incubation. (a) initial sludge; (b) to (d) sludge fed with LJL-2 at 100, 500 and 1000 mg COD/L respectively; (e) to (f) sludge fed with LJL-2 at 100, 500 and 1000 mg COD/L respectively.
(PDF)

S4 Fig SEM images of the bacteria exposed to ZnO nanoparticles. (PDF)
