# Supplementary material for: Biocompatibility and biodegradability of polyacrylate/ZnO nanocomposite during the activated sludge treatment process
Source: PLoS One. 2018 Nov 1;13(11):e0205990. doi: 10.1371/journal.pone.0205990 (PMC6211664; doi:10.1371/journal.pone.0205990)
Supplement: S1 Table — (PDF) [file pone.0205990.s001.pdf]

**S1 Table. Sequence Similarities of Excised DGGE Bands Shown in Fig 5.**

| band No. | closest relative (accession no.) | similarity (%) | taxonomic description |
|---|---|---|---|
| 1 | *Bifidobacterium adolescentis* (NC008618) | 91% | *Actinobacteria* |
| 2 | *Exiguobacterium antarcticum* (NC018665) | 94% | *Firmicutes* |
| 3 | *Cyclobacterium marinum* (NC015914) | 88% | *Unclassified* |
| 4 | *Chitinophaga pinensis* (NC013132) | 83% | *Unclassified* |
| 5 | Flavobacterium johnsoniae (NC009441) | 95% | *Bacteroidetes* |
| 6 | *Dehalococcoides mccartyi* (NC020387) | 86% | *Unclassified* |
| 7 | *Chitinophaga pinensis* (NC013132) | 91% | *Bacteroidetes* |
| 8 | *Frateuria aurantia* (NC017033) | 96% | *Gammaproteobacteria* |
| 9 | *Pseudogulbenkiania* (NC016002) | 96% | *Proteobacteria* |
| 10 | *Anaerolinea thermophila* (NC014960) | 89% | *Unclassified* |
| 11 | *Variovorax paradoxus* (NC014931) | 98% | *Betaproteobacteria* |
| 12 | *Collimonas fungivorans* (NC015856) | 95% | *Betaproteobacteria* |
| 13 | *Ignavibacterium album* (NC017464) | 93% | *Chlorobi* |
| 14 | *Rubrivivax gelatinosus* (NC017075) | 99% | *Betaproteobacteria* |
| 15 | *Nakamurella multipartite* (NC013235) | 92% | *Actinobacteria* |
| 16 | *Rubrivivax gelatinosus* (NC017075) | 96% | *Betaproteobacteria* |
| 17 | *Dechloromonas aromatica* (NC007298) | 98% | *Betaproteobacteria* |
| 18 | *Clostridium stercorarium subsp. stercorarium* (NC020134) | 91% | *Firmicutes* |
| 19 | *Haliscomenobacter hydrossis* (NC015510) | 88% | *Bacteroidetes* |
| 20 | *Clostridiales sp.* (FP929061) | 99% | *Firmicutes* |
| 21 | *Acinetobacter oleivorans* (NC014259) | 94% | *Gammaproteobacteria* |
| 22 | *Paracoccus denitrificans* (NC008687) | 98% | *Alphaproteobacteria* |
| 23 | *Rhodopseudomonas palustris* (NC008435) | 95% | *Alphaproteobacteria* |
| 24 | *Geobacter bemidjiensis* (NC011146) | 86% | *Unclassified* |
| 25 | *Bdellovibrio bacteriovorus* str. *Tiberius* (NC019567) | 91% | *Deltaproteobacteria* |
| 26 | *Legionella pneumophila subsp. pneumophila* (NC018139) | 97% | *Gammaproteobacteria* |
| 27 | *Anaerolinea thermophila* (NC014960) | 91% | *Chloroflexi* |
| 28 | *Pseudomonas putida* (NC018220) | 96% | *Gammaprotebacteria* |
| 29 | *Krokinobacter* sp. (NC015496) | 88% | *Bacteroidetes* |
| 30 | *Hirschia baltica* (NC012982) | 95% | *Alphaproteobacteria* |
| 31 | *Candidatus Nitrospira defluvii* (NC014355) | 100% | *Nitrospirae* |
| 32 | *Segniliparus rotundus* (NC014168) | 92% | *Actinobacteria* |
| 33 | *Ammonifex degensii* (NC013385) | 91% | *Firmicutes* |
| 34 | *Dechloromonas aromatica* (NC007298) | 95% | *Betaproteobacteria* |
| 35 | *Dechloromonas aromatica* (NC007298) | 95% | *Betaproteobacteria* |
| 36 | *Dechloromonas aromatica* (NC007298) | 91% | *Betaproteobacteria* |
| 37 | *Haliscomenobacter hydrossis* (NC015510) | 85% | *Bacteroidetes* |
| 38 | *Thermomonospora curvata* (NC013510) | 88% | *Actinobacteria* |
| 39 | *Sphingobacterium sp.* (NC015277) | 87% | *Bacteroidetes* |
| 40 | *Sorangium cellulosum* (NC010162) | 96% | *Deltaproteobacteria* |
| 41 | *Herminiimonas arsenicoxydans* (NC009138) | 95% | *Betaproteobacteria* |
