# Supplementary material for: Biocompatibility and biodegradability of polyacrylate/ZnO nanocomposite during the activated sludge treatment process
Source: PLoS One. 2018 Nov 1;13(11):e0205990. doi: 10.1371/journal.pone.0205990 (PMC6211664; doi:10.1371/journal.pone.0205990)
Supplement: S2 Table — (PDF) [file pone.0205990.s002.pdf]

S2 Table. Jaccard similarity matrix of bacterial communities in all samples.

	control 0d	control 5d	control 20d	reference 5d	reference 5d	LJL-2H 5d	LJL-2H 20d	LJL-3H 5d	LJL-3H 20d
control 0d	1	0.697	0.6667	0.4444	0.5714	0.5676	0.6286	0.5278	0.6176
control 5d	**0.6970**	1	0.8929	0.5625	0.4324	0.697	0.6667	0.6563	0.6563
control 20d	**0.6667**	**0.8929**	1	0.5806	0.4857	**0.7742**	0.6875	0.7333	0.7333
reference 5d	0.4444	0.5625	0.5806	1	0.5806	0.5758	0.5	0.5313	0.4848
reference 5d	0.5714	0.4324	0.4857	0.5806	1	0.5714	0.5882	0.5294	0.5294
LJL-2H 5d	0.5676	**0.6970**	0.7742	0.5758	0.5714	1	0.8387	0.8966	0.8333
LJL-2H 20d	0.6286	**0.6667**	0.6875	0.5	0.5882	**0.8387**	1	0.8621	0.8
LJL-3H 5d	0.5278	0.6563	0.7333	0.5313	0.5294	**0.8966**	**0.8621**	1	0.7333
LJL-3H 20d	0.6176	0.6563	0.7333	0.4848	0.5294	**0.8333**	0.8	0.7333	1
