# Supplementary material for: Biocompatibility and biodegradability of polyacrylate/ZnO nanocomposite during the activated sludge treatment process
Source: PLoS One. 2018 Nov 1;13(11):e0205990. doi: 10.1371/journal.pone.0205990 (PMC6211664; doi:10.1371/journal.pone.0205990)
Supplement: S3 Table — (PDF) [file pone.0205990.s003.pdf]

S3 Table. Bacterial community diversity of different sludge treatments.

Number	SHANNON(H)	Evenness
Control 0d	4.8574	0.9999
Control 5d	4.7531	0.9996
Control 20d	4.6977	0.9994
Reference 5d	4.519	0.999
Reference 20d	4.6955	0.999
LJL-2H 5d	4.8419	0.9967
LJL-2H 20d	4.7963	0.9977
LJL-3H 5d	4.6908	0.998
LJL-3H 20d	4.6964	0.9991
