# Supplementary material for: Biocompatibility and biodegradability of polyacrylate/ZnO nanocomposite during the activated sludge treatment process
Source: PLoS One. 2018 Nov 1;13(11):e0205990. doi: 10.1371/journal.pone.0205990 (PMC6211664; doi:10.1371/journal.pone.0205990)
Supplement: S4 Table — (PDF) [file pone.0205990.s004.pdf]

S4 Table. Niche width of the main populations observed in incubations.

Species	Levins index
Actinobacteria	7.6165
Bacteroidetes	8.8821
Chlorobi	8.9358
Chloroflexi	7.9818
Firmicutes	7.7218
Nitrospirae	6.9653
Proteobacteria	8.9544
unclassified	8.4287
