# Supplementary material for: Biocompatibility and biodegradability of polyacrylate/ZnO nanocomposite during the activated sludge treatment process
Source: PLoS One. 2018 Nov 1;13(11):e0205990. doi: 10.1371/journal.pone.0205990 (PMC6211664; doi:10.1371/journal.pone.0205990)
Supplement: S5 Table — (PDF) [file pone.0205990.s005.pdf]

S5 Table. Niche overlap of the bacterial populations observed in activated sludge incubations.

	Actinobacteria	*Bacteroidetes*	*Chlorobi*	*Chloroflexi*	*Firmicutes*	*Nitrospirae*	*Proteobacteria*	*unclassified*
Actinobacteria	1	0.8808	0.9195	0.8189	0.9632	0.6573	0.9096	0.8757
Bacteroidetes	0.8808	1	**0.99**	0.9315	0.885	0.8997	0.9921	0.97
Chlorobi	0.9195	0.99	1	0.9256	0.9124	0.8579	0.9961	0.9663
Chloroflexi	0.8189	0.9315	0.9256	1	0.8968	0.9381	0.9277	0.9269
Firmicutes	0.9632	0.885	0.9124	0.8968	1	0.7363	0.9147	0.8555
Nitrospirae	0.6573	0.8997	0.8579	0.9381	0.7363	1	0.8709	0.8936
Proteobacteria	0.9096	**0.9921**	**0.9961**	0.9277	0.9147	0.8709	1	**0.9539**
unclassified	0.8757	0.97	**0.9663**	0.9269	0.8555	0.8936	0.9539	1
