# Supplementary material for: Biocompatibility and biodegradability of polyacrylate/ZnO nanocomposite during the activated sludge treatment process
Source: PLoS One. 2018 Nov 1;13(11):e0205990. doi: 10.1371/journal.pone.0205990 (PMC6211664; doi:10.1371/journal.pone.0205990)
Supplement: S1 Fig — The proportion was calculated by the averages of dehydrogenase and corresponding protein concentration. (PDF) [file pone.0205990.s006.pdf]

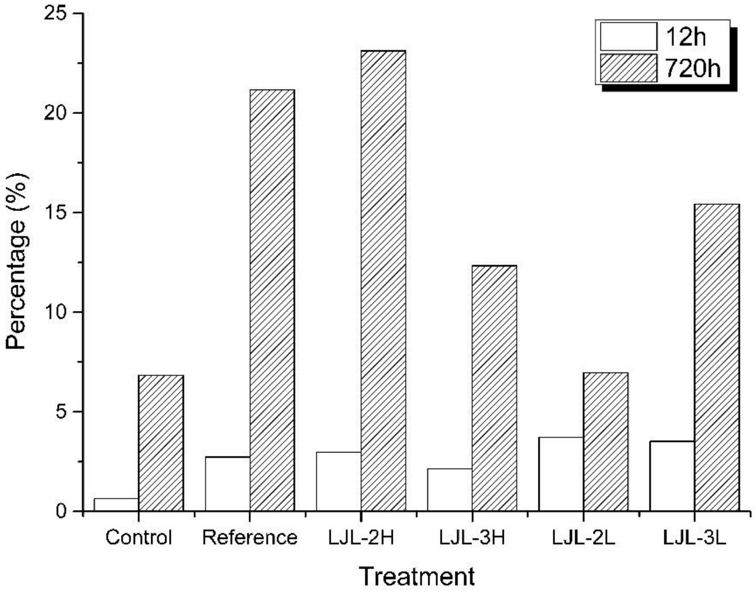

S1 Fig. Comparisons of proportions of dehydrogenase to protein in activated sludge incubations on both 12th and 720th hour. The proportion was calculated by the averages of dehydrogenase and corresponding protein concentration.
