# Supplementary material for: Biocompatibility and biodegradability of polyacrylate/ZnO nanocomposite during the activated sludge treatment process
Source: PLoS One. 2018 Nov 1;13(11):e0205990. doi: 10.1371/journal.pone.0205990 (PMC6211664; doi:10.1371/journal.pone.0205990)
Supplement: S2 Fig — (PDF) [file pone.0205990.s007.pdf]

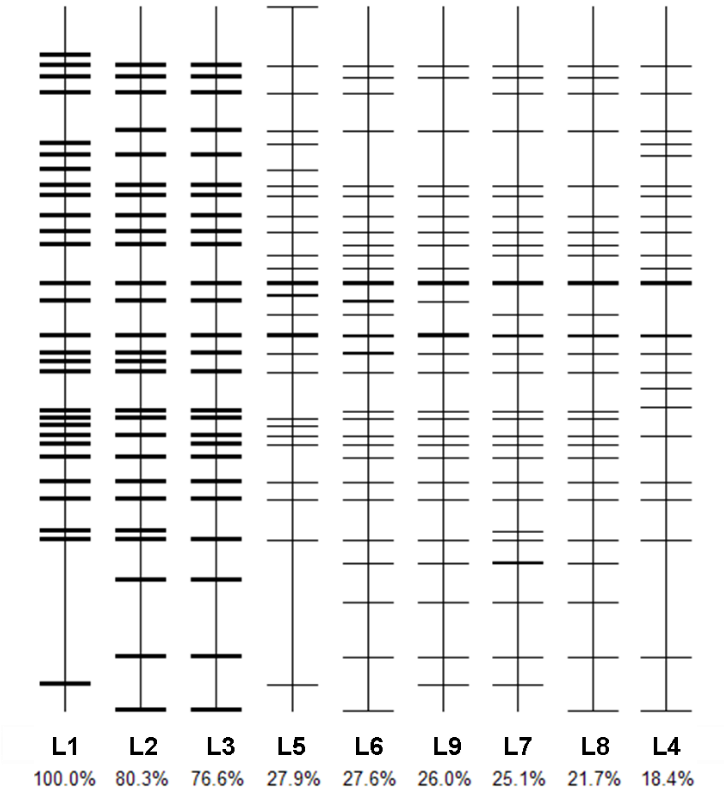

S2 Fig. DGGE fingerprint sketch to order the samples according to their similarities comparing with L1 which represents the bacterial community of original activated sludge.
