# Supplementary material for: Biocompatibility and biodegradability of polyacrylate/ZnO nanocomposite during the activated sludge treatment process
Source: PLoS One. 2018 Nov 1;13(11):e0205990. doi: 10.1371/journal.pone.0205990 (PMC6211664; doi:10.1371/journal.pone.0205990)
Supplement: S3 Fig — (a) initial sludge; (b) to (d) sludge fed with LJL-2 at 100, 500 and 1000 mg COD/L respectively; (e) to (f) sludge fed with LJL-2 at 100, 500 and 1000 mg COD/L respectively. (PDF) [file pone.0205990.s008.pdf]

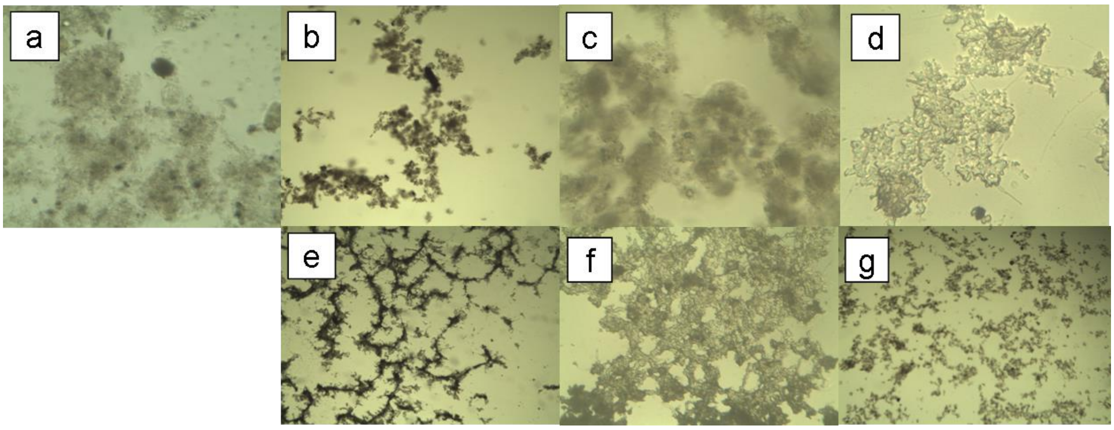

S3 Fig. Microscope images of the sludge treatments after 30-day incubation. (a) initial sludge; (b) to (d) sludge fed with LJL-2 at 100, 500 and 1000 mg COD/L respectively; (e) to (f) sludge fed with LJL-2 at 100, 500 and 1000 mg COD/L respectively.
