# Supplementary figures and images for: Biocompatibility and biodegradability of polyacrylate/ZnO nanocomposite during the activated sludge treatment process
Source: PLoS One. 2018 Nov 1;13(11):e0205990. doi: 10.1371/journal.pone.0205990 (PMC6211664; doi:10.1371/journal.pone.0205990)

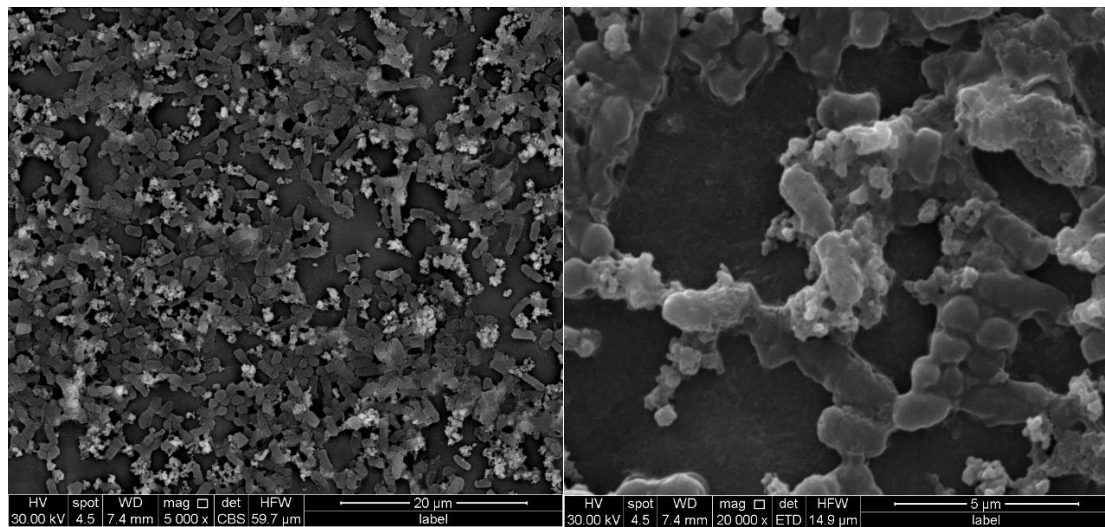

**S4 Fig. SEM images of the bacteria exposed to ZnO nanoparticles.**

Supplement: S4 Fig — (PDF) [file pone.0205990.s009.pdf]
